# An Examination of Forest Certification Status among Logging Companies in Cameroon

**DOI:** 10.1155/2014/323014

**Published:** 2014-12-31

**Authors:** Daniel Nukpezah, Dieudonne Alemagi, Lalisa Duguma, Peter Minang, Charlie Mbosso, Zac Tchoundjeu

**Affiliations:** ^1^Institute for Environment and Sanitation Studies, International House, University of Ghana, 1 Annie Jiagge Road, Legon, Accra, Ghana; ^2^World Agroforestry Centre Regional Office, P.O. Box 16317, Yaoundé, Cameroon; ^3^World Agroforestry Centre, P.O. Box 30677, Nairobi 00100, Kenya

## Abstract

This paper assesses the level of interest, awareness, and adoption of ISO 14001 and Forest Stewardship Council (FSC) certification schemes among logging companies in Cameroon. Eleven logging companies located in Douala in the Littoral Region of Cameroon were assessed through a structured interview using an administered questionnaire which was mostly analyzed qualitatively thereafter. The findings indicated that none of the companies was certified for ISO 14001; however 63.64% of them were already FSC-certified. Four companies (36.36%) were neither FSC- nor ISO 14001 EMS-certified. Among the factors found to influence the adoption rate was the level of awareness about ISO 14001 and FSC certification schemes. The main drivers for pursuing FSC certification were easy penetration into international markets, tax holiday benefits, and enhancement of corporate image of the logging companies through corporate social responsibility fulfillments. Poor domestic market for certified products was found to be the major impediment to get certified. To make logging activities more environmentally friendly and socially acceptable, logging companies should be encouraged to get certified through the ISO 14001 EMS scheme which is almost nonexistent so far. This requires awareness creation about the scheme, encouraging domestic markets for certified products and creating policy incentives.

## 1. Introduction

The attention gained by forest resource use certification has been linked to the fact that natural resources are being exploited unsustainably [1]. A number of efforts have been adopted in an attempt to curb the problem of forest degradation and deforestation, which stands tall among the other global environmental and social concerns [2, 3]. Domestic and allied nongovernmental transnational organizations have engendered private standard setting bodies whose purpose is to recognize officially companies and landowners practicing “sustainable forest management” (SFM) [4]. Furthermore, among governmental mechanisms, voluntary market based tools such as certifications are gaining ground [1].

Between 2000 and 2003 alone, the area of certified forests worldwide doubled to approximately 150 million hectares [5]. There are many certification schemes; however this study focuses on those of the Forest Stewardship Council (FSC) and the International Standardization Organization (ISO) 14001 environmental management system (EMS) standard which is the most common and widely recognized EMS certification. Both ISO 14001 EMS and FSC certification schemes have been hailed as contributors to sustainable forest management [6]. Simply put, ISO 14001 EMS is an environmental management system (EMS) which ensures “a transparent, systematic process known corporate-wide, with the purpose of prescribing and implementing environmental goals, policies, and responsibilities, as well as regular auditing of its elements” [7]. On the other hand, the Forest Stewardship Council certification seeks to support environmentally appropriate, socially beneficial, and economically viable management of the world's forests [8]. In this paper, we present the status of both certification schemes among logging companies in Cameroon and suggest possible recommendations for improving their implementation among these companies.

## 2. Theoretical Framework

An environment management system (EMS) based on the requirements of ISO 14001 is a management tool that enables an organization of any type or size in identifying and controlling the environmental impacts of its activities, continually improving its environmental performance, implementing a systematic approach in setting environmental objectives, and demonstrating that such objectives have been successfully achieved [9, 10]. In effect, ISO 14001 entails the commitment of management and employees for the protection of environment with clear assignment of accountability and responsibility [11].

FSC's principal activities are geared towards the development of forest management and related performance standards, communications, and education and, through a separate programme, the accreditation and monitoring of certification bodies working to facilitate FSC performance standards [12]. FSC is the only certification system that is most widely recognized and with wide geographic distribution in terms of forest management. The success as well as stringency of the FSC provides a comparative framework for the successful implementation of product certification as a means of forestry conservation [13]. Indeed, this may account for the growing interest in the uptake of FSC certification schemes globally. FSC certification is based on 10 principles that are applicable to tropical forests including [14]

- compliance with country laws and international legislation;
- a clear definition, documentation, and legal establishment of long-term tenure and user rights;
- identification and endorsement of the rights of indigenous people with regard to ownership and use of land and resources;
- maintenance or enhancement of the socioeconomic wellbeing of forest workers and local communities;
- maintenance or enhancement of the economic, social, and environmental benefits from the forest on a long-term basis;
- maintenance or restoration of the ecosystem, its biodiversity, resources, and landscapes.

Although both ISO 14001 and FSC aim at sustainable forest management (SFM), it is of merit to highlight some differences. The International Standardization Organization [10] points out that EMS covers similar ground to forest management certification, except that it does not specify forest management performance standards and does not permit a label to be attached to products [10]. Thus while FSC is set up as an international accreditation body for forest management certification and labeling, for ISO 14001 EMS, it is the management system that is certified, and not the forest itself. Further it has been asserted that despite being not strictly a forest certification programme, the ISO 14001 approach offers much potential for assessing the environmental quality of forest management [15].

In addition, while both FSC and the ISO 14001 aim to improve environmental performance, they are otherwise very much different in terms of structure and operation. The FSC and ISO approaches have two distinct philosophies with respect to forest certification for logging companies—the former emphasizing forest performance standards and the latter management system standards [15]. In spite of the distinction between the two approaches, there is considerable overlap between them in many matters as well as the kind of communication that exists between them [15]. It must be emphasized that while ISO 14001 EMS and FSC certifications have had different degrees of success in several jurisdictions globally, not much is known in terms of its adoption rate and socioeconomic and environmental impacts amongst logging companies in Cameroon. This study therefore seeks to

- assess the prevailing levels of adoption, interest, and awareness of ISO 14001 and FSC certification among logging companies in Cameroon,
- explore the socioeconomic and environmental implications resulting from the implementation of ISO 14001 and FSC certifications by these companies.

## 3. ISO 14001 EMS and FSC Certification Processes

In order to be ISO 14001-certified, a company must first demonstrate that it has developed and implemented an EMS. An EMS typically includes organizational procedures, environmental responsibilities, and processes that can help organizations such as logging companies comply with environmental regulations, identify technical and economic benefits, and ensure that corporate environmental policies are adopted and followed [16]. The most common and widely recognized EMS certification is ISO 14001 which is awarded by ISO accredited certification bodies. ISO 14001 EMS requires that specific procedures are put in place for environmental monitoring, assessment, and measurement purposes.

The process for developing and implementing an EMS especially ISO 14001 EMS is the drafting of an environmental policy. The corporate environmental policy outlines the environmental objectives of the logging company. The environmental policy serves to inform the community and the company's employee as well as the other stakeholders about the company's intentions with respect to the environment. The prepared document must be documented, implemented, maintained, and communicated to all workers.

Next, the EMS must have a documented planning process that identifies all relevant “environmental aspects” along with the existing environmental strengths and weaknesses of the organizations activities, products, and processes as well as applicable legal and regulatory requirements. Additionally, specific environmental objectives and targets must be highlighted in relation to how the company intends to respond to current and anticipated environmental issues (impacts) from both technological and administrative perspectives [17]. In other words the company must set out an environmental management program.

Further the environmental responsibilities of all levels of the workforce must be assigned and external stakeholder parties and general groups must be informed of corporate goals and strategies. At this stage also, the company proceeds to determine and develop necessary procedures and commit whatever resources are needed to implement the corporate environmental strategy.

The EMS action plan is principally a task for top management. It involves top management identifying pertinent environmental objectives and actions and conducting multiple environmental reviews before implementing the EMS. Development of an environmental reporting scheme is important at this stage [17].

Next step is evaluation. Here the primary purpose is to assess the company's actual environmental performance against the stated environmental policies, objectives, and targets that comprise the EMS action plan. Feedback from evaluation helps to improve corporate procedures and processes. Evaluation is accomplished using the rudimentary auditing strategies, or by using more advanced monitoring and control [17].

Fourth and final stage is corrective action. Where environmental performance has not met what has been stipulated in the environmental policies, reviews are done and corrective actions are put in place to remedy the shortfall.

In order to achieve certification for an established EMS, however, an ISO accredited auditor (external consultant) is brought in for assessment. The auditor compares the company's documented system with the requirements of the ISO 14001 standard. Documentation must be approved before certification can be granted. The auditor normally conducts the document review on site, but this may be conducted off site as well. There is usually an ISO 14001 audit checklist given to the company in advance to aid its preparation for the document review.

The certification assessment itself has two main stages. The first stage involves determining the company's state of readiness and defining the approach and duration of the second stage. During this first stage, the lead auditor would finalize the document review, execute a facility inspection, review the environmental permits applicable to the organization, and review the organization's identified environmental aspects and impacts. This stage involves a limited number of personnel, including the management representative (http://en.wikipedia.org/wiki/Forest_Stewardship_Council).

The second stage, on the other hand, assesses the extent of implementation and effectiveness of the environmental management system. The ISO 14001 auditor executes with the aim of documenting with objective evidence the effectiveness of the system through an extensive review of records and interview of significant portion of the employees at all levels of the organization (http://en.wikipedia.org/wiki/Forest_Stewardship_Council).

At the end of the assessment the accredited auditor makes recommendation whether or not the company should receive certification. If certification is granted, the company is provided with ISO 14001 certificate and appropriate organizations are notified of the company's successful certification.

Forest management certification is a voluntary process for verifying responsible forest practices. It is to the discretion of a forest owner or representative of a group of forest owners and operators to initiate the certification process by requesting an accredited independent certifier to inspect the forest and to see if the management meets the Forest Stewardship Council's requirements for certification (http://en.wikipedia.org/wiki/Forest_Stewardship_Council).

The process involves the submission of an application by a forest manager or landowner to an accredited certifier. The certifier then conducts an audit of the forest to be certified by assessing the quality of its management against the Forest Stewardship Council's requirements for certification encapsulated in the council's principles and criteria (P&C). The certifier's report is usually reviewed by recognized experts or peers and if the forest under consideration meets the requisite standards, a certificate is issued and the forest becomes certified against a specific standard [18].

Continuous holding of the certificate is however subject to review by an FSC accredited certification body. This is done through annual compliance review and a comprehensive audit in a prespecified period [18]. Certificate holders are charged annual fees to review their accreditation. Only where continuous compliance is established can certificate holders keep their certificates.

## 4. Literature Review

Both the theoretical literature and empirical literature provide some insight into ISO 14001 and FSC certification schemes globally. However, the vast majority of research on ISO 14001 implementation at the corporate level has occurred in developed economies. For example, it has been shown that the implementation of ISO 14001 by the oil and gas industry in the United Kingdom resulted in improved environmental performance and increased awareness of environmental issues by employees at the corporate level [19]. Another study revealed that there was low uptake of ISO 14001 amongst small and medium size enterprises engaged in timber preservation in developed countries due to the lack of the requisite finance to fund the cost associated with the implementation of ISO 14001 [20]. Studies in Spain pointed to improvement in corporate image as a result of implementing ISO 14001 [21]. In a study of Japanese firms it was concluded that a reduction in the risk of accident at the corporate level was a fundamental achievement emanating from the implementation of ISO 14001 [22].

With respect to FSC certification on the African continent, a study was conducted to find out why uptake of FSC chain of custody certification remains low among export timber firms in Ghana [23]. It was realised that the absence of political commitment, the lack of clarity in the objectives of certification, land tenure issues, lack of resources, difficulty in the recognition of national schemes, poor communication, and low literacy levels were the principal constraints to the adoption of this certification scheme in Ghana [23]. Another study posits that, for several years now, forestry companies in Central Africa (considered here as Cameroon, Equatorial Guinea, Democratic Republic of Congo (DRC), Congo, Central African Republic (CAR), and Gabon) are increasingly engaged in a quest for a more sustainable forest harvesting by developing integrated management plans [24]. This has resulted in approximately 30 million hectares of dense rain forests under management or engaged in the process of developing a management plan [24]. In a study [25], it was discovered that despite the many positive perceptions of FSC certification it emerged from interviews that certification was yet to make any headway amongst many Community-Based Forest Operations (CFOs) in Cameroon. Similar to these arguments, there is the view that many logging companies continue to exploit Cameroon's rain forests because nobody was willing to pay for the conservation of the country's forest ecosystems [26]. An evaluation of land tenure security revealed that insecurity of land tenure places a considerable obstacle on the adoption of ISO 14001 and FSC certification in Cameroon, as indigenous logging companies and CFOs do not have permanent land ownership within community forests because their customary land rights within these forests are not constitutionally or legally protected [26].

From the literature review, while there has been extensive commentary on ISO 14001 and FSC certification schemes and especially with regard to CFOs, no empirical assessment has been made in terms of levels of interest, adoption, and socioeconomic and environmental impacts for large-scale forest concessions (logging companies) in Cameroon. Indeed, while all of the reviewed studies are instructive, academic studies on the impacts of ISO 14001 and FSC implementation by these logging companies, for example, remain lacking. Against this background, we argue that this study would make a significant positive contribution to the literature on ISO 14001 and FSC certification as schemes that contribute towards sustainability.

## 5. Methodology

In this study, the authors make use of overwhelmingly qualitative research approach. However, the study also makes use of basic quantitative tools like computing percentages and averages. A two pronged research approach was followed for this study. First, the authors sought information from the secondary literature. Such sources included relevant governmental legislation and reports, working papers from government and civil society, and peer-reviewed literature. Analysis of secondary literature helped with a systematic review and evaluation of these data sources as they related to logging companies and helped shape the themes presented in this paper. Secondly, primary data was obtained through interview with eleven logging companies based in the Douala city located in the Littoral Region of Cameroon. Doula city became the target of our study because it is the main port at which timber and logs from the country's forests are processed and exported to the international market. The sample size was limited to eleven only due to the fact that many logging companies are hesitant to release corporate information to the public and we decided to emphasize only on the eleven companies that were willing to provide the necessary information required for the study.

A questionnaire, which was the research instrument, was developed to elicit responses from logging companies on various aspects of their operations, including certification for both ISO 14001 EMS and FSC certification; reasons preventing logging companies from seeking certification; perceived benefits associated with certification; motivations for certifications; social and economic impacts of ISO 14001 certification and challenges experienced by logging companies in seeking ISO 14001 EMS certification. We targeted managers of the logging operations as interviewees as we thought such individuals could ably represent the logging companies operations strongly. All the approached interviewees consented to participate in the study.

Interviews were conducted between May and June 2013 during field visits. The interviews were structured, meaning that well-defined questions were prepared in advance by means of a questionnaire seeking the interviewee to respond to specific questions. The questions were also a mix of open ended and close ended questions. These two approaches in our view provided the balance that this research work seeks. For example, structured questions allow the respondent to stay within a defined course while open ended questions allow the interviewer to explore and tap other responses the researcher might not have thought of. Both of these approaches were used to achieve the research objectives especially since our research work is partly exploratory.

Questionnaires were administered by trained field assistants to eleven registered logging companies. In all, all the eleven logging companies responded within a time frame of 2 days average after the questionnaire was first administered. In assessing the information gathered, the transcribed interviews were analyzed qualitatively by selecting relevant passages or quotes from the transcripts. After an initial open-coding process [27] these were then coded according to emergent themes identified.

## 6. Results and Discussion

### 6.1. ISO 14001 and FSC Certification Status of Selected Cameroonian Logging Companies

Among the selected eleven logging companies none was certified for the ISO 14001 EMS while 63.64% (seven companies) were already FSC-certified. However, one company (WIJMA Cameroon) has done a preaudit for ISO 14001 EMS certification. Four companies were neither FSC- nor ISO 14001 EMS-certified. However all these four noncertified logging companies expressed interest in getting certified in the future. The level of interest for certification among these four noncertified companies can therefore be said to be high. The fact that almost two-thirds of the companies are FSC-certified versus none of them getting ISO 14001 EMS-certified implies strong interest of the logging companies in the former than in the latter.

### 6.2. Factors Impeding the Certification of the Logging Companies in the Cameroon Context

Indeed per the responses obtained, among the reasons impeding company's move for certification is the poor demand for certified products in the domestic market. This assertion suggests that, from the respondents' perspectives, the market available for certified forest goods was less than noncertified forest goods partly due to the higher prices of the former compared to the latter due to the costs associated with the certification scheme. Thus profit oriented companies may not see the added value for certification except adding costs of production particularly if their market focus is domestic. ISO and FSC certifications in developing nations most at times generate considerable problems in the search for markets for their products because demand for certified timber in local markets is generally low [28, 29]. Thus, depending on the market, and to the extent that logging companies find local markets not demanding certified wood, they may not be compelled to seek certification. As one respondent firm (TRANSIBOIS) put it:

*“In making a comparative report between investments and what comes out (produce), we have no interest since the volume of certified products demanded is small and negligible compared to what we are producing.”*

Another noncertified company (TJK) however pointed out that it is still collecting data to launch the certification process. Yet again another company pointed out that it is a new company (SEPBC) and does not have certified products to sell. From the foregoing, it may be argued that limited products and limited market for certified products seem to be the overarching reason for some of the companies not seeking certification.

### 6.3. Awareness of the Benefits of ISO 14001 and FSC Certifications among Noncertified Companies

It is worth noting that many of the noncertified companies (for both ISO 14001 and FSC) showed some awareness of the benefits of certification. For example, to a question whether the noncertified companies know of any benefits associated with FSC and ISO 14001 certifications, many provided interesting perspectives. A respondent (TJK) asserts that certification provides adequate means for the traceability of wood. Indeed, the respondent representing this company added that he hopes to find time and attend certification seminars so as to better understand. This admission may well suggest that the respondent has only a vague understanding of the benefits of certification and does not have demonstrable knowledge of the benefits of certification. This underscores the importance of knowledge and awareness creation for ISO 14001 and FSC certification to gain acceptance amongst logging companies in Cameroon.

A second respondent (TRANSIBOIS) mentioned that “FSC is important in setting up environmental projects as well as takes into consideration the social and environmental aspects of sustainable development.” The respondent further stated that “ISO 14001 on its part is proentrepreneurial since its logic is based on a continuous improvement of the enterprise.” This response somewhat suggests some knowledge of the theoretical foundations of both ISO 14001 and FSC certifications. Although it is true that ISO 14001 is based on continuous improvement of the enterprise, this relates primarily to the environmental aspect of the firms' activities.

A third respondent firm (SEPBC) revealed that “FSC is good for American and European markets” suggesting that FSC may be an attractive marketing imperative for companies that seek to penetrate those markets in the logging industry. This company (SEPBC) further asserts that FSC takes into consideration the environment, health, security, and social aspects of the neighboring population. Indeed, this assertion is consistent with the principles espoused by the Forest Stewardship Counsel [14] which include “maintenance and enhancement of the socioeconomic wellbeing of forest workers and local communities” [14]. In addition, this company (SEPBC) stated that FSC demands systematic or annual inventories, restoration of the site, fight against poaching, and the development of activities with the neighboring population (Forest Peasant Committee). Again, this assertion is in agreement with one of the principles of the FSC scheme that applies to tropical forests. This principle endorses the “maintenance or restoration of the ecosystem, its biodiversity, resources, and landscapes” [14]. Thus, there seems to be advanced knowledge by this company about the reasons underpinning the need for certification and the awareness of benefits and this may suggest that although not certified yet, this company has found the need to read the literature on certification or has been attending certification seminars.

Finally a fourth company (ALPICAM) posited that FSC certification results in “better management of the environment as ISO 14001 covers the environment in general while FSC concerns mostly forest management.” Further, according to this company (ALPICAM), FSC enhances the image of the company and it is a more easy way of setting up a “green policy” and that in the long run the company can benefit from tax reductions (since the legal framework relative to environmental management makes provisions for this). It is worthy to note that this company did not attribute enhancement of corporate image to certification of ISO 14001 as well although one of the benefits of ISO 14001 as reported in the literature includes enhancement of corporate image [30, 31]. Indeed this assertion by the respondent suggests a greater knowledge and awareness of the benefits of FSC certification compared to ISO 14001.

It is instructive that while the question posed relates to knowledge of the benefits of both ISO 14001 and FSC certifications, the responses were skewed more towards FSC certifications. This suggests that while knowledge as well as awareness of the benefits of FSC certification is high, same conclusion cannot be drawn about ISO 14001 certification among noncertified companies. This may well explain the generally high uptake of FSC certification (63.64%) among the companies studied compared to zero uptake (although one company is at a preaudit stage) for ISO 14001. We conclude here that the level of awareness and interest in certification is higher for FSC certification than ISO 14001 certification. Undeniably, numerous studies [15, 32, 33] have identified that, economically, quite a number of logging companies with forest licenses in Cameroon have limited knowledge of ISO 14001 and FSC certification due to the fact that they lacked the necessary funds needed to implement forest certification. Our study on the other hand showed relatively high knowledge of FSC certification. This may well be because awareness of the benefits of forest certification has increased since these works were first published. It is also an important pointer to the fact that a conscious effort was made to have increased FSC certification schemes. Indeed, it has been reported that much of the support for forest certification has been the result of larger retailers whose corporate images have been threatened by environmental NGO campaigns and much of the certification uptakes may be related to pressure or endorsement by such NGOs [5]. It is important to note that ISO 14001 certification has not received such attention in terms of endorsement by such NGOs. While this may also partly explain the zero uptake of ISO 14001 certification among logging companies in Cameroon, our analysis clearly suggests that relatively low levels of knowledge and awareness are major culprits.

### 6.4. Motivations and Benefits of Certification among FSC Certified Logging Companies

Many of the respondents touted the drivers and benefits of FSC certification and explained their motivations for getting certified. Ease of market penetration is a compelling driver for seeking certification. All (100%) FSC certified firms agreed that easy access to international markets was a prevailing driver. As one company asserts, certification is in “response to the needs of our principal market: that is, Holland.” Four (4) out of seven (7) of the FSC certified logging companies surveyed were of the view that FSC certification is an important marketing tool and was one of the reasons why they got certified. Indeed, there is no gainsaying that the Forest Stewardship Council (FSC) forest certification is broadly regarded as a marketing tool that is used by forest owners and logging companies who perceive an economic benefit from undergoing the certification (FAO Forest Resources Assessment 2000, cited in [6]).

Another important reason for seeking certification is for ecological benefits. Six out of the seven (85.71%) FSC certified companies agreed that they are driven by ecological protection. For some forest certified companies, endorsing forest certification provides them with the satisfaction of supporting the sustainability of natural forest resources and more broadly society at large [34] and as such this may also serve to improve their corporate images and access to markets [35, 36]. In connection to this, adoption of forest certification may be driven, in part, by firms being ethically and morally motivated to integrate environmental and social aspects into their marketing plans [37, 38]. Over 70% representing five (5) out of seven (7) respondent firms cited enhancement of corporate image as a motivation for seeking certification. The findings also reveal that, among the FSC certified logging companies, two out of seven firms assigned legislative reasons and financial benefits such as guaranteeing of tax holidays as a compelling reason for seeking certification. None of the companies saw increased corporate profitability as a reason for getting certified.

In addition to the above, respondents provided other additional benefits for getting certification. One respondent firm agrees that it “receives a large financial value on certified tropical wood in European markets.” Sustainable forest management gives assurance that the wood is not stolen but has been legally obtained from a certified forest was one of the other reasons given for seeking certification. Further, one of the respondents explained that “certification is an endorsement of the authenticity of the wood products and brands the wood as coming from a sustainably managed forest.” In fact, per the findings of this study, there are other flanker benefits and drivers associated with certification. For example, the standardized practice associated with certification extends “social and security benefits to workers and it provides a platform for best labor contracts; medical check-ups; provision of work-clothing for laborers, and the like” as put by one respondent. Thus, here the respondents have emphasized the economic and social impacts of FSC certification per the objective of the study.

Some of the logging companies asserted that requirement such as forest companies should be “strict in the work quality and quantity as per the norms of their enterprise and ensuring that personnel management as well as its impact on the environment is considered” endeared them to take up certification. Chronicling further the benefits and other drivers of FSC certification, it was revealed by one logging company that FSC “changes its practices to new practices which responds to social, environmental, and biodiversity needs; it respects laws and regulations in terms of salaries and commercial guarantee of wood. It also ensures traceability of wood products when this becomes important.” Further, another motivation for seeking certification gleaned from this study is that some of the respondents see the label “FSC” as something they could leverage to give them competitive advantage. As one of the companies put it:

*“We are able to show that we are carrying out a responsible activity and the label ‘FSC' gives us a certain level of prestige over others.” *

Again one certified company contends that certification serves as an entry barrier since it limits competition. Indeed entry barriers protect incumbent firms from potential entrants [39] and this lends credence to the view that certification can be used to reduce threats by new entrants. Finally, FSC certification seems to have a demand side benefit of scale effect [39]. Demand side benefits of scale arise in industries where a buyer's willingness to pay for a product increases with the number of other buyers who also patronize the company's product [39]. Customers may trust certified logging companies making such companies have more market share. Competitors may thus want to go for certification too if they can afford it in order not to lose their buyers. Indeed as one respondent put it: “certification is the most current in forestry and in Cameroon these days.” This may well suggest that some companies seek certification because it is fashionable and because other firms within the industry are pursuing this path, if they are not seen pursuing similar path, they might lose out.

## 7. Conclusion and Recommendations

This study has shown that the level of interest, awareness, and adoption of FSC certification is high among logging companies in Cameroon. On the other hand, we have argued that the level of interest, awareness, and adoption of ISO 14001 is mostly low among the logging companies and in some cases inconclusive. Regarding the impacts arising from implementation, those impacts could not be determined for ISO 14001 certification since none of the companies were certified for it. On the other hand, the socioeconomic and environmental impacts resulting from the implementation of FSC certification include financial benefits such as guaranteeing of tax holidays, ecological benefits such as an endorsement of the authenticity of the wood products and branding the wood as coming from a sustainably managed forest, and extension of social and security benefits to workers such as providing a platform for best labor contracts, medical check-ups, and the provision of work-clothing for laborers. Further, this study has shown that, among others, the drivers for seeking FSC certification are entry access to international markets and ecological benefits. The study therefore suggests that using environmental/ethical attributes and access to international markets as a mechanism to promote uptake of FSC certification could be an important marketing imperative towards achieving sustainable forest management.

Again we identified factors such as low levels of knowledge and awareness regarding ISO 14001 certification as contributing to the significant lack of interest in adopting certification. Regarding FSC certification, the level of knowledge and awareness is high and to the extent that logging companies find markets for certified products, they are inclined to seek certification. It is our considered view that potential to develop markets for ISO 14001 certified wood products exists if efforts are made to improve awareness of ISO 14001 certification benefits. Finally while we have conjectured reasons for logging companies not seeking ISO 14001 certification, it is our view also that the reasons why the respondent logging companies prefer and take up only FSC certification should be researched. We note that in this study none of the companies have been certified for both ISO 14001 and FSC certification schemes. Indeed it is certainly of academic value to explore the possibilities, potentials, and drivers for dual certification, their implications, and benefits. This would provide a more rigorous basis for managing tropical forests sustainably. We therefore recommend that future studies should also research into this.
